# Alcohol consumption among tertiary students in the Hohoe municipality, Ghana: analysis of prevalence, effects, and associated factors from a cross-sectional study

**DOI:** 10.1186/s12888-021-03447-0

**Published:** 2021-09-03

**Authors:** Richard Gyan Aboagye, Nuworza Kugbey, Bright Opoku Ahinkorah, Abdul-Aziz Seidu, Abdul Cadri, Paa Yeboah Akonor

**Affiliations:** 1 Department of Family and Community Health, School of Public Health, University of Health and Allied Sciences, Ho, Ghana; 2 Department of General Studies, University of Environment and Sustainable Development, Somanya, Ghana; 3 School of Public Health, Faculty of Health, University of Technology Sydney, Sydney, Australia; 4 College of Public Health, Medical and Veterinary Services, James Cook University, Douglas, Australia; 5 Department of Social and Behavioural Science, School of Public Health, University of Ghana, Legon-Accra, Ghana

**Keywords:** Alcohol consumption, Risk factors, Perceived effects, Tertiary students, Ghana

## Abstract

**Background:**

Alcohol consumption constitutes a major public health problem as it has negative consequences on the health, social, psychological, and economic outcomes of individuals. Tertiary education presents students with unique challenges and some students resort to the use of alcohol in dealing with their problems. This study, therefore, sought to determine alcohol use, its effects, and associated factors among tertiary students in the Hohoe Municipaility of Ghana.

**Methods:**

An institutional-based cross-sectional study was conducted among 418 tertiary students in the Hohoe Municipality of Ghana using a two-stage sampling technique. Data were collected using structured questionnaires. A binary logistic regression modelling was used to determine the strength of the association between alcohol consumption and the explanatory variables. The level of significance was set at *p* < 0.05. Stata version 16.0 was used to perform the analysis.

**Results:**

The lifetime prevalence of alcohol consumption was 39.5%. Out of them, 49.1% were still using alcohol, translating to an overall prevalence of 19.4% among the tertiary students. Self-reported perceived effects attributed to alcohol consumption were loss of valuable items (60.6%), excessive vomiting (53.9%), stomach pains/upset (46.1%), accident (40.0%), unprotected sex (35.1%), risk of liver infection (16.4%), depressive feelings (27.3%), diarrhoea (24.2%), debt (15.2%), and petty theft (22.4%). In terms of factors associated with alcohol consumption, students aged 26 years and above were more likely to have consumed alcohol [AOR = 4.4, 95%CI = 1.74, 11.14] than those in 16–20 years group. Muslim students had lower odds of alcohol consumption compared to Christians [AOR = 0.1, 95% CI = 0.02, 0.31]. It was also found that students who had peer influence [AOR = 3.7, 95%CI = 2.31, 5.82] and those who had academic adjustment problems [AOR = 3.6, 95% CI = 2.01, 6.46] were more likely to consume alcohol.

**Conclusion:**

Lifetime prevalence of alcohol consumption is high among tertiary students in the Hohoe Municipality of Ghana, with several physical, psychosocial and economic consequences. Alcohol-related education should be intensified in tertiary institutions and counseling units should be equipped with relevant assessment tools to assess and help students who are at risk and those who are already consuming alcohol.

**Supplementary Information:**

The online version contains supplementary material available at 10.1186/s12888-021-03447-0.

## Background

Alcohol consumption is an integral part of many cultural, religious, and social practices worldwide [1, 2]. However, in recent years, the volume and risky pattern of consumption pose a significant public health threat to the consumer, family, friends, and the larger society [3–5]. Harmful alcohol consumption results in health, social, and economic burden on both the individual and others in both immediate and distal environments [4].

Alcohol is a commonly used substance among the youth in tertiary institutions [5, 6]. In many instances, alcohol serves as a gateway to the usage of other psychoactive substances [6]. Tertiary education is a transitional period characterized by autonomy or independence from family control, and self-decision making, academic pressures, forming social groups, and sharing living quarters with new students with diverse cultural values [5, 7–9], and peer influence [9–13]. Other factors shown by researchers to predispose students to alcohol consumption include; ease of availability and accessibility of alcohol [14], academic pressures [12, 15], family member use of alcohol [8], and psychological distress [3, 16]. These features of tertiary institutions’ environment elsewhere are synonymous with those in the Ghanaian setting [17].

Globally, alcohol consumption is the leading causal factor for the overall morbidity and mortality burden [18, 19]. Harmful alcohol consumption serves as a risk factor in over 200 diseases and injuries [4]. These diseases and injuries contribute to about 3million deaths annually, representing 5.3% of all mortality globally and 132.6 million (5.1%) disability-adjusted life years (DALYs) [2, 4]. About 13.5% of all mortality cases in young people aged 20–39 years were attributed to excessive alcohol consumption [2]. However, the association between alcohol consumption and its negative health implications remains complex and inconclusive given the protective effects of moderate alcohol consumption on the human body [19].

Alcohol consumption during the early years is associated with negative consequences such as alterations in attention, verbal learning, and memory, along with altered development of major parts of the brain [20]. These negative consequences subsequently lead to behavioural, emotional, social, and academic problems in later life [21]. Researchers have shown that harmful alcohol consumption leads to the development of cardiovascular diseases [18], cancer [22], liver diseases [23], hepatitis [24], risky sexual behaviours and sexually transmitted diseases [25, 26], mental and behavioural disorders, injuries, violence [2], and poor academic performance [27–29].

Most countries in sub-Saharan Africa are experiencing rapid economic, social, and cultural transitions which have created an avenue for increased and socially disruptive use of alcohol [30]. Ferreira-Borges et al. [31] asserted that alcohol consumption and disease burden in Africa are expected to increase, but that policymakers have paid little or no attention to the issue of increasing alcohol consumption. Studies conducted in various parts of Africa reported a significant prevalence of alcohol consumption among tertiary students. For instance, reported lifetime and current prevalence of alcohol consumption ranged from 16.9 to 34.5% in Ethiopia [8, 9, 32], 31.1 to 78.4% in Nigeria [33, 34], 31.9% in Botswana [5], 50.7–63.2% in Kenya [35, 36], and 2.7% in Sudan [37]. 

Limited studies (example [17, 38]) have been conducted on alcohol consumption among tertiary students in Ghana. This makes it difficult to implement effective interventions due to the dearth of literature on the magnitude of alcohol consumption and its contributory factors. In Ghana, recent evidence showed that there has been an increase in the promotion, competition, and popularity of alcohol beverages in both the media and non-media sources [38]. These alcoholic beverages are considerably cheaper than soft drinks. As a result, young people (majority of which are students) tend to consume alcoholic beverages due to its accessibility and low cost [38]. Anecdotally, there has been an increased proliferation of drinking spots, night clubs, and alcoholic vending sites in the Hohoe Municpality. This has also resulted in easy accessibility to alcoholic beverages by students in the Hohoe Municipality. Hence, the present study sought to determine the prevalence of alcohol consumption and its associated factors among tertiary students in the Hohoe Municipality of Ghana. The findings are likely to inform the development of school-based programmes and interventions aimed at reducing alcohol consumption and promoting healthy lifestyles among students.

## Methods

### Study setting

We conducted the study in the Hohoe Municipality, which is one of the seventeen (17) administrative municipalities/districts in the Volta region. It shares borders with the Republic of Togo on the East, Afadjato district on the Southeast, south by Ho Municipality, Southwest by Kpando Municipality, Northwest by Biakoye district, and on the North with Jasikan district [39]. According to the 2010 Population and Housing Census, the municipality has a population of 167, 016 with a growth rate of 2.5%. Females make up 52.1% of the entire district population whilst males represent 47.9% [39]. The district has a total land area of 1172 km^2^. In terms of education, 0.8% of the inhabitants in the municipality are in tertiary institutions [39]. Tertiary institutions in the municipality include; the University of Health and Allied Sciences (School of Public Health-UHAS), Midwifery Training School (MTS), Saint Theresa’s Training College (THERESCO), and Saint Francis Training College (FRANCO).

### Study design

Institutional-based cross-sectional study was conducted among tertiary students in the Hohoe Municipality using the quantitative technique. Tertiary students from three (3) institutions were recruited for the study. Students on short courses or sandwich programmes, absent on the day of data collection, and sick or  had difficulty to communicate were excluded from the study. We relied on the strengthening the reporting of observational studies in epidemiology statement writing the manuscript.

### Sample size determination and sampling procedure

The sample size for the study was determined using the Cochran formula; n = $\frac{{\mathrm{z}}^2\mathrm{x}\ \mathrm{p}\ \left(1-\mathrm{p}\right)}{{\mathrm{d}}^2}$ [40]. Where n = required sample size, z = reliability coefficient (z-score) of 1.96 at 95% confidence level, p = estimated proportion who use alcohol, and d = margin of error of 5% (0.05). With a 44.9% prevalence of alcohol consumption among students in Cape Coast Metropolis [41] and a 10% non-response rate, the estimated sample size for the study was 418 tertiary students.

A two-stage sampling technique was used to recruit the 418 tertiary students. A simple random technique was first used to select three tertiary institutions using ballotery without replacement method. The three schools that were randomly selected were UHAS, MTS, and FRANCO.

In the second phase, we employed a proportionate stratified sampling method to apportion the sample size per each institution based on the students’ population size. We calculated the sample size for each school as the product of the total students’ population in a selected school and the total sample size for the study divided by the total students’ population in the three schools. With a total students population of 2001 from the three selected schools as at the time of the study, the calculated sample size per each selected school was UHAS (125), MTS (65), and FRANCO (228).

At the school level, a simple random sampling technique using the lottery method was used to recruit the students to include in the study. Pieces of paper with inscription “YES” or “NO” written on them were used and the students were asked to pick one each. Any student who picked “YES” was given a consent form and both parental/guardian consent and assent forms to those below 18 years for their voluntary approval to take part in the study. We repeated the procedure in all selected schools until we obtained the required sample size.

### Data collection procedure

A self-administered structured questionnaire was used to collect data from the students. We developed the questionnaire from a review of pertinent literature on the subject [34–36]. Detailed questionnaire has been attached as a supplementary file. We pretested the developed questionnaire among 42 tertiary students who were not part of the actual study in the Hohoe Municipality. We then administered the pretested questionnaire to the students with the aid of five (5) trained data collectors. We structured the questionnaire into four (4) sections. These sections comprised the sociodemographic characteristics, the pattern of alcohol consumption, psychosocial factors of alcohol consumption, and perceived effects of alcohol consumption. We included students aged 18 years and above who signed the written informed consent. For those below 18 years, we sought written informed consent from their parents or guardians and child assent form from the student. Both written parental or guardian informed consent and child assent forms were required before the students aged below 18 years were included in the study and given a questionnaire to complete.

### Statistical analyses

Data collected from the respondents were entered into EpiData 3.1 and exported into Stata version 16.0 for the analysis. The exported data were cleaned, validated, and coded for analysis. We presented categorical variables using frequencies and percentages in tables and charts. A Chi-square test was first performed to determine the relationship between lifetime alcohol consumption and the explanatory variables. We performed a binary logistic regression analysis to determine the strength of association between lifetime alcohol consumption and the explanatory variables. All the variables that showed statistical significance were placed in the regression model. We presented the results of the regression analysis using crude odds ratio (COR) and adjusted odds ratio (AOR) with their corresponding confidence interval (CIs) and *p*-value. A *p* < 0.05 was considered statistically significant, showing the level of precision.

### Ethical issues

We obtained ethical approval for the study from the Ghana Health Service Ethics Review Committee (GHS-ERC) with a reference number (GHS-ERC:92/10/16). We strictly adhered to the ethical guidelines and protocols put forth by the GHS-REC throughout the study. We also sought institutional approval from the Ghana Education Service and Municipal Health Directorate, Hohoe, and Heads of the various institutions. Before the commencement of data collection, written informed consent was sought from students aged 18 years and above before inclusion in the study. For students aged below 18 years, written informed consent was obtained from each student’s parents or guardian before participating in the study. Additionally, written parental or guardian consent and child assent were sought from each student before inclusion in the study. All ethical issues concerning research among humans were strictly followed. Respondents’ rights to withdraw from the study, confidentiality, participants’ privacy, risk, and benefits involved in the study were duly explained to the students after which interested respondents voluntarily signed the written consent or assent forms.

## Results

### Sociodemographic characteristics of the tertiary students

Of the 418 tertiary students, 51.4% were males. The majority (65.3%) of the students were aged 21–25 years with the mean age of 22.4 ± 3.1 years. Almost all the students (95.0%) were single. Most of the students were Christians (87.1%) and residents on school campuses (64.6%). In the year of study, 39.0% were in the first year as shown in Table 1.

**Table 1:** Sociodemographic characteristics of the tertiary students

Variable	Frequency (***n*** = 418)	Percentage (%)
**Mean age of students (years ± SD)**	**22.4 ± 3.1 years**	
**Age group**		
16–20	111	26.6
21–25	273	65.3
26 and above	34	8.1
**Sex**		
Male	215	51.4
Female	203	48.6
**Marital status**		
Single	397	95.0
Married	21	5.0
**Institution**		
UHAS	125	29.9
FRANCO	228	54.5
MTC	65	15.6
**Religion**		
Christian	364	87.1
Traditional	8	1.9
Muslim	46	11.0
**Year(s) of study**		
One	163	39.0
Two	115	27.5
Three	101	24.2
Four	39	9.3
**Residential status**		
Resident	270	64.6
Non resident	148	35.4

### Pattern of alcohol consumption among the tertiary students

The lifetime prevalence of alcohol consumption was 39.5%. Out of them, 49.1% were still using alcohol, translating to an overall current prevalence of 19.4% among the tertiary students. The majority (83.0%) of alcohol consumers started between the ages of 16–20 years. The mean age of alcohol initiation was 18.9 ± 2.7 years. Seventy-two (43.6%) of the students consume alcohol yearly. Beer (37.6%) was the most consumed alcoholic beverage followed by wine (35.2%). On a typical day, 41.2% of the students consume alcohol 1–2 times. Also, 28.5% of the students drunk alcohol at least once in the past week prior to the study as presented in Table 2.

**Table 2:** Pattern of alcohol consumption among the students

Variables	Frequency (n)	Percentage (%)
**Ever consumed alcohol (n = 418**)		
Yes	165	39.5
No	253	60.5
**Current users of alcohol (*n*** **= 165)**		
Yes	81	49.1
No	84	50.9
**Mean age of initiation (years ± SD)**	18.9 ± 2.7 years	
**Age of initiation**		
10–15	9	5.5
16–20	137	83.0
21–25	15	9.1
26 and above	4	2.4
**Frequency of alcohol consumption**		
Daily	9	5.5
Weekly	36	21.8
Monthly	48	29.1
Yearly	72	43.6
**Type of alcoholic beverage consumed**		
Beer	62	37.6
Wine	58	35.2
Spirits	9	5.5
Gin	10	6.1
All	26	15.8
**Number of drinks per typical day**		
None	91	55.2
1–2 times	69	41.2
3 and above	5	3.6
**Number of drinks in the past week**		
None	98	59.4
Once	47	28.5
Twice	14	8.5
Three or more	6	3.6

### Psychosocial factors of alcohol consumption

**Fig. 1:**
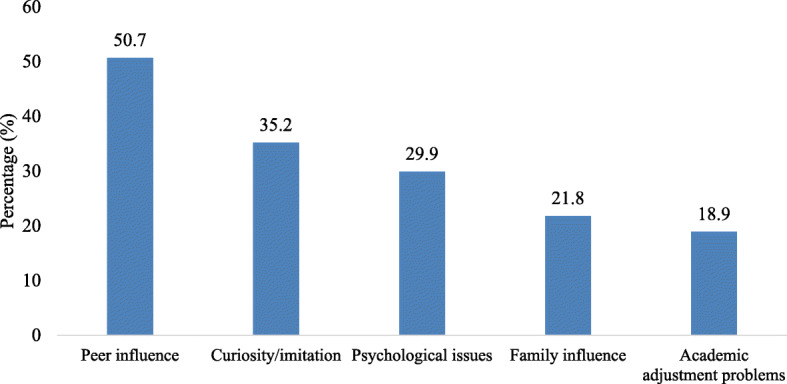
Psychosocial factors influencing alcohol consumption

Two hundred and twelve respondents (50.7%) attributed alcohol consumption among students to peer influence. Regarding curiosity/imitation, 35.2% of the respondents reported curiosity/imitation to influence students’ alcohol consumption. Also, psychological issues (29.9%), family influence (21.8%), and academic adjustment problems (18.9%) were some of the key reasons influencing alcohol consumption among tertiary students as presented in Fig. 1.

### Factors influencing alcohol consumption among tertiary students

Results from the bivariate analysis (chi-square analysis) showed that age group (χ^2^ = 13.16, *p* < 0.001), sex (χ^2^ = 10.43, p < 0.001), religion (χ^2^ = 27.90, *p* < 0.001), peer influence (χ^2^ = 47.17, p < 0.001), and academic adjustment problems (χ^2^ = 28.31, p < 0.001) were significantly associated with alcohol consumption among tertiary students (Table 3).

**Table 3:** Bivariate analysis of factors associated with alcohol consumption among tertiary students

Variable	Alcohol consumption		Chi-square (χ^2^)	P-value
	No (253)	Yes (165)		
**Age group**			13.16	< 0.001
16–20	78 (70.3)	33 (29.7)		
21–25	168 (61.5)	105 (38.5)		
26 and above	11 (32.4)	23 (67.6)		
**Sex**			10.43	< 0.001
Male	114 (53.0)	101 (47.0)		
Female	139 (68.5)	64 (31.5)		
**Marital status**			1.54	0.214
Single	243 (61.2)	154 (38.8)		
Married	12 (52.2)	11 (47.8)		
**Religion**			27.90	< 0.001
Christian	203 (55.8)	161 (44.2)		
Traditional	6 (75.0)	2 (25.0)		
Muslim	44 (95.7)	2 (4.3)		
**Year of study (s)**			7.58	0.055
**One**	107 (65.6)	56 (34.4)		
**Two**	65 (56.5)	50 (43.5)		
**Three**	64 (63.4)	37 (36.6)		
**Four**	17 (43.6)	22 (56.4)		
**Residential status**			0.29	0.589
Resident	166 (61.5)	104 (38.5)		
Non-resident	87 (58.8)	61 (41.2)		
**Peer influence**			47.17	< 0.001
No	159 (77.2)	47 (22.8)		
Yes	94 (44.3)	118 (55.7)		
**Curiosity/imitation**			0.39	0.533
No	167 (61.6)	104 (38.4)		
Yes	86 (58.5)	61 (41.5)		
**Psychological issues**			2.80	0.094
No	185 (63.1)	108 (36.9)		
Yes	68 (54.4)	57 (45.6)		
**Family influence**			1.42	0.233
No	193 (59.0)	134 (41.0)		
Yes	60 (65.9)	31 (34.1)		
**Academic adjustment**			28.31	< 0.001
No	226 (66.7)	113 (33.3)		
Yes	27 (34.2)	52 (65.8)		

### Predictors of alcohol consumption among tertiary students

Results of the regression analysis of predictors of alcohol consumption among tertiary students has been shown in Table 4. In the adjusted model, students aged 26 years and above were 4.4 times more likely to consume alcohol compared to those aged 16–20 years, and the association was statistically significant [AOR = 4.4, 95% CI = 1.74, 11.14]. Muslim students were 90.0% less likely to consume alcohol compared to their Christian counterparts [AOR = 0.1, 95% CI = 0.02, 0.31]. Students with peer influence had higher odds of alcohol consumption as against those without peer influence [AOR = 3.7, 95% CI = 2.31, 5.82]. Also, students with academic adjustment problems were more likely to consume alcohol compared to their counterparts without academic problems [AOR = 3.6, 95% CI = 2.01, 6.46].

**Table 4:** Logistic regression analysis of predictors of alcohol consumption among tertiary students

Variables	COR (95% CI)	AOR (95% CI)
**Age group**		
16–20	1.0	1.0
21–25	1.3 [0.79. 1.99]	1.7 [0.99, 2.82]
26 and above	4.2* [1.84, 9.49]	4.4* [1.74, 11.14]
**Sex**		
Male	1.0	1.0
Female	0.5* (0.35, 0.77)	0.9 [0.54, 1.34]
**Religion**		
Christian	1.0	1.0
Traditional	0.4 [0.08, 2.11]	0.3 [0.04, 2.03]
Muslim	0.1** [0.01, 0.24]	0.1** [0.02, 0.31]
**Peer influence**		
No	1.0	1.0
Yes	4.2** [2.78, 6.49]	3.7** [2.31, 5.82]
**Academic adjustment problems**		
No	1.0	1.0
Yes	3.8** [2.30, 6.47]	3.6** [2.01, 6.46]

COR = Crude Odds Ratio; AOR = Adjusted Odds Ratio, 1 = Reference * = ***p*** < 0.01 ** = p < 0.001

### Perceived effects of alcohol consumption

Commonly reported effects attributed to alcohol consumption among the students were loss of valuable items (60.6%), and excessive vomiting (53.9%) as shown in Table 5.

**Table 5:** Perceived effects of alcohol consumption among the tertiary students

Variable	Frequency (n = 165)	Percentage (%)
**Excessive alcohol consumption can cause diarrhoea**		
Agree	40	24.2
Disagree	125	75.8
**Excessive alcohol consumption can cause stomach upset/pain**		
Agree	76	46.1
Disagree	89	53.9
**Can increase one’s chances of getting an accident**		
Agree	66	40.0
Disagree	99	60.0
**Can cause a bloated stomach**		
Agree	134	20.6
Disagree	131	79.4
**Can cause depressive feeling for weeks**		
Agree	45	27.3
Disagree	120	72.7
**Can cause one to vomit excessively**		
Agree	89	53.9
Disagree	76	46.1
**Serves as a risk factor for liver infection**		
Agree	27	16.4
Disagree	138	83.6
**Influence one to engage in unprotected sex**		
Agree	58	35.1
Disagree	107	64.9
**Can cause one to lose money and valuable items**		
Agree	100	60.6
Disagree	65	39.4
**Can cause one to incur debts**		
Agree	25	15.2
Disagree	140	84.8
**Can cause one to engage in petty theft**		
Agree	37	22.4
Disagree	128	77.6

## Discussion

This study sought to determine the prevalence of alcohol consumption among tertiary students in the Hohoe Municipality of Ghana. We also assessed the factors associated with alcohol consumption and the perceived effects of alcohol among the consumers. We found that the overall lifetime and current prevalence of alcohol consumption among the students were 39.5% and 19.4% respectively. These findings are similar to that of Gebremariam et al. [32] who reported the lifetime and current alcohol consumption prevalence of 36.3% and 16.9% respectively among university students in Ethiopia. However, our prevalence rates were lower than some studies from Kenya [35], Nigeria [6, 33], and Ghana [17, 38]. For instance, the study conducted in Ghana reported an ever alcohol consumption prevalence of 56.3%, whiles current consumers were 25.8% [17]. Also, Hassan [36] found in Kenya that lifetime alcohol consumption was prevalent among 63.2% of tertiary students. The observed differences in prevalence could be because of the inclusion of two health tertiary institutions in the current study. These students from health institutions might be knowledgeable about the health implications of alcohol consumption hence the less consumption rate.

Our findings showed that the odds of alcohol consumption among the students increased with increasing age. Students aged 26 years and above were more likely to consume alcohol. This finding is consistent with results from a cross-sectional study conducted in China [42] and  Nigeria [33]. The finding also confirms the association between older age and alcohol consumption found in a study that used data from 24 different countries [43]. The plausible explanation of the finding could be that older students were more likely to access alcohol because they have passed the legal age of alcohol consumption [33]. Also, older age is associated with societal pressure, stress, and increased quest to achieve success and this could have increased their likelihood of resorting to consuming alcohol as a way of coping [33].

Consistent with previous literature from Ethiopia [7, 9] and Ghana [38], this study found that being a Muslim was associated with lower odds of alcohol consumption. This finding is not surprising as alcohol consumption is prohibited in the Islamic religion. It is against the religious doctrines as a Muslim to consume alcohol.

Also, the study found that peer influence was associated with higher odds of alcohol consumption. This finding corroborates studies from South Africa [13], Ethiopia [8, 9, 12, 15, 44] where peer influence was a significant predictor of alcohol consumption among tertiary students. Studies conducted in Ghana also reported similar findings [10, 11]. The social learning principle which emphasizes that individuals can learn bad behaviours from watching their peers [14] could explain the findings in the study. Also, as peers are important sources of social support and therefore, their pressure can be an enforcer for good and bad behaviour [14].

We found academic adjustment problems to be a significant predictor of alcohol consumption among the students. This finding is in line with previous studies which reported significant associations between students’ academic adjustment problems and alcohol consumption [12, 15, 45]. That is, the rate of alcohol consumption was higher among students with academic problems or those dissatisfied with their academic performance. Plausible factors that could explain the observed association include; difficulties in balancing academics with social life, low level of commitment towards the field of study, and course and assignment overload which could have predispose the students to consume alcohol.

### Limitations of the study

The cross-sectional nature of the study did not allow for inferences of causality between alcohol consumption and the risk factors to be made. Second, the self-reported pattern of alcohol consumption and the perceived effects of alcohol use are often prone to recall and social desirability biases. Also, the study cannot be generalized to the general population because of the involvement of only tertiary students. Additionally, we did not perform rigorous statistical analysis for the perceived effects of alcohol consumption among the students. Furthermore, sample weights were not used in the present study and this limits it’s generalizability to other tertiary students. The use of simple random sampling technique in selecting study schools is another limitation of the study given the varying students population in the selected schools.

## Conclusion

Our study found a relatively high prevalence of alcohol consumption among tertiary students in the Hohoe Municipality. Almost half of lifetime alcohol consumers were current drinkers. Among the lifetime alcohol consumers, self-reported effects included stomach pains or upset, accident, unprotected sex, loss of valuable items, excessive vomiting, diarrhea, risk of liver infection, debt, and petty theft. Factors perpetuating alcohol consumption among the students were peer pressure, increasing age (26 years and above), and academic adjustment problems. Being a Muslim was protective against alcohol consumption. Regular alcohol assessment should be carried out in tertiary schools to help identify students with potential alcohol problems in order for appropriated and tailored psychosocial interventions. Students with poor academic performance and psychological distress should be counseled to help them cope with their challenges without resorting to alcohol consumption. Health education on alcohol consumption, the risk factors and its effects should be intensified especially in both health and non-health training institutions highlighting the short- and long-term consequences of alcohol and the role of peers in shaping their behaviour.

## Supplementary Information

**Additional file 1.** Questionnaire.

## Data Availability

The datasets used or analysed during the current study are available from the corresponding author on reasonable request.

## Abbreviations

AOR: Adjusted Odds Ratio
CI: Confidence Interval
COR: Crude Odds Ratio
DALYs: Disability Adjusted Life Years
FRANCO: Saint Francis Training College
GHS-ERC: Ghana Health Service Ethics Review Committee
MTS: Midwifery Training School
THERESCO: Saint Theresa’s Training College
UHAS: University of Health and Allied Sciences
