# Supplementary material for: Alcohol consumption among tertiary students in the Hohoe municipality, Ghana: analysis of prevalence, effects, and associated factors from a cross-sectional study
Source: BMC Psychiatry. 2021 Sep 3;21:431. doi: 10.1186/s12888-021-03447-0 (PMC8414877; doi:10.1186/s12888-021-03447-0)
Supplement: Supplementary file 1 — Additional file 1. Questionnaire. [file 12888_2021_3447_MOESM1_ESM.pdf]

Alcohol consumption among tertiary students in the Hohoe Municipality, Ghana: Analysis of prevalence, effects and associated factors from a cross-sectional study

Richard Gyan Aboagye[1], Nuworza Kugbey[2], Bright Opoku Ahinkorah[3], Abdul-Aziz Seidu[4], Abdul Cadri[5], Paa Yeboah Akonor[1]

QUESTIONNAIRE

SECTION A: Socio-demographic characteristics

	**QUESTIONS**	**RESPONSE**
00	Respondent code	………………….
01	Respondent institution	………………….
1.	What is your sex?	1. Male<br>2. Female
2.	What is your age in complete years?	…………………
3.	What is your resident status)?	1. Resident<br>2. Non-resident
4.	What is your marital status?	1. Single<br>2. Married
5.	What is your religious affiliation?	1. Christian<br>2. Muslim<br>3. Traditionalist
6.	What is your year(s) of study?	1. One<br>2. Two<br>3. Three<br>4. Four
	**SECTION B: Pattern of alcohol consumption**	
7.	Have you ever in your lifetime taken any alcoholic beverage before?	1. Yes<br>2. No (Skip to Q15)
8.	Do you currently drink alcoholic beverages?	1. Yes<br>2. No
9.	At what age did you start drinking alcoholic beverages?	………………….
10.	How often do you consume alcoholic beverages?	1. Daily<br>2. Weekly<br>3. Monthly<br>4. Yearly
11.	Which of the alcoholic beverages do you drink?	1. Beer<br>2. Wine<br>3. Spirits<br>4. All<br>5. Others, please specify ………...
12.	Currently, how many times do you take alcoholic drinks on a typical day?	1. 1-2 times<br>2. 3-5 times

		3. 5 or more times<br>4. None
13.	How many times did you take alcohol in the past week?	1. Not at all<br>2. Once<br>3. Twice<br>4. Three or more times
	**SECTION C: Factors influencing alcohol consumption**	
14	Why do students consume alcohol?	
14.1	Peer influence	1. Yes<br>2. No
14.2	Family influence	1. Yes<br>2. No
14.3	Curiosity	1. Yes<br>2. No
14.4	Academic adjustment	1. Yes<br>2. No
14.5	Psychological issues	1. Yes<br>2. No
14.6	Others, specify	……………….

SECTION D: Consequences of alcohol consumption (Only those who consume alcohol)

	STATEMENT	Agree	Disagree
	**Effects of alcohol use on academic performance**		
15	Alcohol use affects students' academic performance		
16	Alcohol use among students increases absenteeism		
17	Alcohol use among students increases lateness to class		
18	Alcohol use increases low participation in class		
19	Alcohol use affects student's inability to complete assignments		
	**Economic effects of alcohol use**		
20	I have lost money and other valuable items as a result of alcohol use		
21	I sometimes pawn belongings to buy alcohol		
22	I sometimes engage in petty theft to gain money in order to buy alcohol		
23	I have several debts to pay for consuming alcohol		
	**Health effects**		
24	I do experience diarrhoea when I consume alcoholic drinks		
25	I do experience stomach upset/pain after drinking alcohol		
26	I have been involved in an accident that requires hospital treatment due to alcohol consumption		
27	I have developed a bloated stomach since I started drinking		
28	I do feel depressed for more than a week after consuming alcohol		
29	I usually engage in unprotected sex after drinking alcohol		
30	I do vomit after excessively consuming alcohol		
31	I have been diagnosed of infection to my liver since I started drinking		
32	I have experienced weight loss since I started consuming alcohol		

THANK YOU
